# VIS Atlas: A Database of Virus Integration Sites in Human Genome from NGS Data to Explore Integration Patterns

**DOI:** 10.1016/j.gpb.2023.02.005

**Published:** 2023-02-16

**Authors:** Ye Chen, Yuyan Wang, Ping Zhou, Hao Huang, Rui Li, Zhen Zeng, Zifeng Cui, Rui Tian, Zhuang Jin, Jiashuo Liu, Zhaoyue Huang, Lifang Li, Zheying Huang, Xun Tian, Meiying Yu, Zheng Hu

**Affiliations:** 1 Department of Obstetrics and Gynecology, the First Affiliated Hospital, Sun Yat-sen University, Guangzhou 510000, China; 2 Department of Obstetrics and Gynecology, Dongguan Maternal and Child Health Care Hospital, Dongguan 523000, China; 3 Office of Scientific Research & Development, Sun Yat-sen University, Guangzhou 510000, China; 4 Department of Obstetrics and Gynecology, Academician Expert Workstation, The Central Hospital of Wuhan, Tongji Medical College, Huazhong University of Science and Technology, Wuhan 430000, China; 5 Center for Translational Medicine, the First Affiliated Hospital, Sun Yat-sen University, Guangzhou 510000, China; 6 Department of Pathology, the Central Hospital of Enshi Tujia and Miao Autonomous Prefecture, Enshi 445000, China; 7 Department of Obstetrics and Gynecology, Zhongnan Hospital of Wuhan University, Wuhan 430062, China

**Keywords:** DNA virus, Virus integration site, Next-generation sequencing, Integration pattern, Virus genotype

## Abstract

Integration of oncogenic **DNA viruses** into the human genome is a key step in most virus-induced carcinogenesis. Here, we constructed a **virus integration site** (VIS) Atlas database, an extensive collection of integration breakpoints for three most prevalent oncoviruses, human papillomavirus, hepatitis B virus, and Epstein–Barr virus based on the **next-generation sequencing** (NGS) data, literature, and experimental data. There are 63,179 breakpoints and 47,411 junctional sequences with full annotations deposited in the VIS Atlas database, comprising 47 **virus genotypes** and 17 disease types. The VIS Atlas database provides (1) a genome browser for NGS breakpoint quality check, visualization of VISs, and the local genomic context; (2) a novel platform to discover **integration patterns**; and (3) a statistics interface for a comprehensive investigation of genotype-specific integration features. Data collected in the VIS Atlas aid to provide insights into virus pathogenic mechanisms and the development of novel antitumor drugs. The VIS Atlas database is available at https://www.vis-atlas.tech/.

## Introduction

Viral infections contribute to approximately 10%–15% of human cancer burden [1], causing 1.6 million new malignancies annually [2]. The integration of oncogenic viruses into the human genome is an important step to induce carcinogenesis [3]. The insertional events may induce some negative effects on host cells. First, integration induces genomic instability and generates mutations in key cancer-associated genes, providing opportunities for the malignant transformation of infected cells [4], [5]. Second, the integrated viral elements could function as strong *cis*-activators of nearby oncogenes to promote tumorigenesis [6]. Third, viral integration could produce virus–human fusion transcripts/proteins that may act as carcinogenic drivers, conferring host cells additional selective advantages in transformation [5], [7]. These roles of DNA virus integrations make them attractive targets for early prevention and therapeutic intervention. However, despite the biological importance, the integration patterns and mechanisms of different viruses and specific viral genotypes are still poorly understood.

Traditionally, viral integration can be detected using fluorescence *in situ* hybridization [8], amplification of papillomavirus oncogene transcript assay [9], or polymerase chain reaction (PCR)-based methods [10]. However, data generated using these methods are often low-throughput or biased. In recent years, next-generation sequencing (NGS) technologies are becoming a popular approach for virus integration detection. In addition, various virus enrichment strategies and diverse bioinformatic tools have been developed, such as VirusSeq [11], ViralFusionSeq [12], VirusFinder [13], HIVID [14], Virana [15], Virus-Clip [16], VERSE [17], Vy-PER [18], ViFi [19], and VirTect [20]. As so far, NGS data generated massive virus integration sites (VISs) [21], [22], [23]. However, they had different sensitivities, resolutions, display forms, genome versions, and quality assessment systems. Meanwhile, current known virus integration databases, Dr.VIS v2.0 [24], HPVbase [25], and VISDB [26] merely provided the collection of virus integration positions from different original studies, most of them without single-base human–virus junctional sequences. For instance, VISDB only deposited junction sequences of less than 5 percent of VISs (n = 1615) [26]. Furthermore, a comprehensive comparison between VIS Atlas and VISDB was carried out from different aspects (Table S1). Obviously, VIS Atlas and VISDB are quite different in virus compositions, genotypes, data resources, and sample types. Besides, VIS Atlas has provided an integration pattern illustrating tool, many more single-base resolution breakpoints, and more details on the distribution of VISs, which could help study the integration-triggered local mutations and design-targeted genome editing tools, and understand the mechanism of oncogenic virus integration. Therefore, a universal and sensitive collection of VISs in single-base resolution is necessary and remains a great challenge.

Our team has successfully developed a VIS detection pipeline algorithm (VIPA) [27], [28], [29], [30]. Based on VIPA, we presented the VIS Atlas database, an extensive collection of human–virus breakpoints for three most prevalent oncovirus, human papillomavirus (HPV), hepatitis B virus (HBV), and Epstein–Barr virus (EBV). Generally, 77.28% of breakpoints (n = 48,828) of our database were derived from NGS data with the rest 22.72% (n = 14,351) coming from literature and experimental data. Altogether, the VIS Atlas database provided 63,179 accurate breakpoints (HPV: 36,145; HBV: 25,616; and EBV: 1418), covering 47 virus genotypes and 17 disease types. To our knowledge, VIS Atlas is the largest DNA virus integration database to date.

## Data collection and processing

### System design and implementation

The general workflow of VIS Atlas was developed by MySQL (version 5.7.24; https://www.mysql.com/). The VIS Atlas was designed and the interactive interface was built using Vue (version 2.6.10; https://cn.vuejs.org/index.html) and Ant Design (version 1.3.10; https://ant.design/index-cn). The ECharts (version 4.2.1; https://echarts.apache.org) was used as a graphical visualization framework, and JBrowse (version 1.16; http://jbrowse.org) was used as the browser framework. We recommend using the database with a modern web browser that supports HTML5, such as Firefox, Google Chrome, Safari, Opera, or IE 10.0+. VIS Atlas is freely available to the research community, and users are not required to register or login to access information in the database.

### Data collection

Two kinds of data sources were involved in this study, computational breakpoints and curated breakpoints. For NGS-based computational breakpoints, the raw data collection included three databases. (1) The Cancer Genome Atlas (TCGA) database: under the TCGA-CESC project, we downloaded all 615 bam files with sequencing reads (Data Category) and whole-exome sequencing (WES) (Experimental Strategy) filter conditions for all 307 cases. The download activities were finished by binary Genomic Data Commons (GDC) Data Transfer Tool, gdc-client from GDC Data Portal (https://portal.gdc.cancer.gov/) with the authorization of Sun Yat-sen University. (2) The Sequence Read Archive (SRA) database: all raw sequencing data were retrieved by statements of virus full names, virus abbreviations, full names of virus-related cancers, or abbreviations of virus-related cancers, and then filtered by public source and DNA strategy. All metadata of the aforementioned results were examined to exclude the runs of epigenomics strategy, experimental intervened samples, or the third-generation sequencing platform. Then, runs of 2043 samples were downloaded by the Linux wget command. (3) The European Bioinformatics Institute (EBI) database: some candidate data searched from SRA were deposited and only could be accessed in EBI. (4) In-house samples: all 6075 cervical exfoliative cell samples, Raji cell line samples, and C666.1 cell line samples from Zheng Hu Lab were conducted with virus capture technology accompanied by NGS. For literature/experiment-validated breakpoints, data from Dr.VIS v2.0 were downloaded before they were inactive, and 11 papers were kept with integration sequences for curation. Publications were also searched from PubMed and Google Scholar with the authorization of Sun Yat-Sen University by the statements of virus integration, virus full names, or virus abbreviations. Some papers already employed in Dr.VIS v2.0 were checked for accuracy and completeness. Then, we kept 25 papers, which were not employed in Dr.VIS v2.0 otherwise with correct information. We also detected HPV breakpoints for 397 samples by detection of integrated papillomavirus sequences by ligation-mediated PCR (DIPS-PCR) technology.

### Detection of VISs and sequences

Two kinds of data sources were processed in different ways. For NGS data, the soft-clip and discordant reads are the main evidence of virus integration, and the former provides accurate integration information. Therefore, we developed the bioinformatic pipeline VIPA based on soft-clip reads to detect VISs and assemble integration sequences.

**Figure 1:**
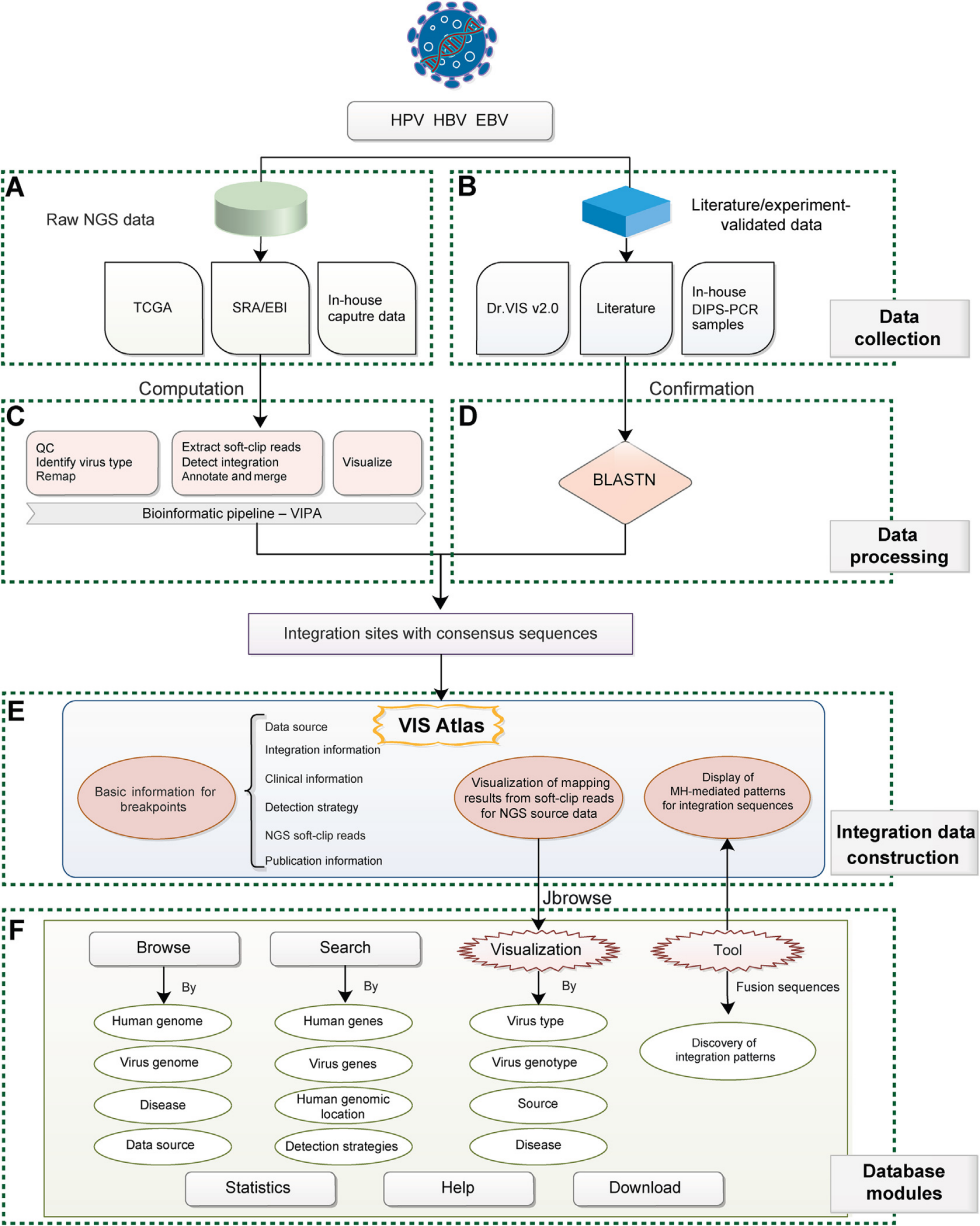
**Overview of VIS Atlas** Data mainly came from NGS databases, as well as literature/experiments, followed by different processing methods. Full genome and source annotations of each breakpoint are included. **A.** Source of NGS data. **B.** Source of literature/experiment-validated data. **C.** VIPA, the computational pipeline of VISs for NGS data. **D.** Confirmation of VISs for literature/experiment-validated data. **E.** Integration data models for each breakpoint. **F.** Seven modules in the VIS Atlas database. HPV, human papillomavirus; HBV, hepatitis B virus; EBV, Epstein–Barr virus; TCGA, The Cancer Genome Atlas; SRA, Sequence Read Archive; VIS, virus integration site; EBI, European Bioinformatics Institute; PCR, polymerase chain reaction; DIPS-PCR, detection of integrated papillomavirus sequences by ligation-mediated PCR; QC, quality control; NGS, next-generation sequencing; VIPA, VIS detection pipeline algorithm; MH, microhomology.

The related steps were listed below (Figure 1**)**. (1) Quality control (QC): quality of all collected raw WES data was tested by FastQC followed by simple QC by fastp [31]. (2) Reference preparation: human genome reference GRCh38.p12 was downloaded from the University of California Santa Cruz (UCSC; http://genome.ucsc.edu/). Representative virus genome references were downloaded from the Papillomavirus Episteme (PaVE) genome database (http://pave.niaid.nih.gov), Hepatitis B Virus Database (https://hbvdb.lyon.inserm.fr/HBVdb/; Accession number: AB014381.1, AB032431.1, AB033554.1, AB036910.1, AB064310.1, AF090842.1, AY090454.1, M32138.1, NC_003977.2, X02763.1, and X51970.1), and the National Center for Biotechnology Information [https://www.ncbi.nlm.nih.gov/; Type 1 (NC_007605) and Type 2 (NC_009334)]. (3) Virus infection identification: BWA-MEM was used to map clean data to the mixed reference of human and all viruses to identify the dominant infection virus type. (4) Remap: clean data were mapped to (i) mixed reference of human and detected virus genotype; (ii) human reference; and (iii) detected virus genotype reference by BWA-MEM, followed by removing duplication by SAMtools (samtools v-0.1.19) and Picard MarkDuplicates (Picard tools v-1.117) command. (5) Soft-clip read extraction: soft-clip reads, defined as reads spanning the junction sites of human and virus genomes (pair-end soft-clip reads and one-end soft-clip reads), were extracted based on the aforementioned mapping results. These reads were re-aligned against the human reference, respectively, and virus reference by BLASTN (BLAST v-2.7.1). Only reads with consistent alignment results of BWA and BLAST were retained for the next step. (6) Human–virus breakpoint identification: the junction positions of soft-clip reads were merged according to the junction positions in both human and virus genomes, and supported soft-clip reads were calculated for each position. (7) Annotation and consensus sequence generation: junction positions were annotated by ANNOVAR (version 2017-07-17) for the human genome and in-house scripts for the virus genome. For EBV, the VISs located in the repeat region of the EBV genome were excluded. Then, we conducted multiple alignments of supported soft-clip reads by ClustalW, and used EMBOSS Cons to generate consensus junctional sequences. Finally, detailed breakpoint information that met the filtering standard (soft-clip reads ≥ 2) for HPV, HBV, and EBV was generated. (8) The mapping results of soft-clip reads were extracted for manual visualization.

For literature/experiment-validated breakpoints, including the Dr.VIS v2.0 database, updated publications, and DIPS-PCR-detected VISs, the sequencing results were collected followed by BLASTN against the unified human and virus genome references to curate the positions and filter unreliable ones in data processing. By this method, we kept 201, 268, and 354 breakpoints of the Dr.VIS v2.0 database, updated publications, and DIPS-PCR-detected VISs, respectively.

## Database content and usage

### Database overview

Overview of the VIS Atlas database is shown in Figure 1. There were two main data sources making up the VIS Atlas database: (1) NGS data from TCGA, SRA/EBI database, and virus capture data of in-house samples (Figure 1A); and (2) literature/experiment-validated data (Figure 1B). We processed the aforementioned two kinds of data, respectively. The NGS data were analyzed by VIPA bioinformatic pipelines to identify VISs and consensus sequences (Figure 1C). Literature/experiment-validated integration sequences (literature, Dr.VIS v2.0 database, and in-house DIPS-PCR experiments) were curated by mapping to human and virus genome references via BLASTN (Figure 1D). Then, we built a three-level VIS Atlas model for each integration item (Figure 1E): (1) basic information, including data source, integration information, clinical information (disease, pathology, and stage), detection strategy, NGS reads, and publication information; (2) visualization of supporting reads for NGS-derived computational breakpoints in VIS Browser; and (3) display of sequence results with details of microhomology (MH)-mediated patterns. For VIS Atlas database construction, we designed seven modules, including Browse, Search, Visualization, Tool, Statistics, Help, and Download (Figure 1F).

### High-resolution virus integration data

In the VIS Atlas database, we constructed a universal dataset of VISs for oncogenic viruses. Altogether, 63,179 accurate VISs involved in three most prevalent DNA oncoviruses (HPV, HBV, and EBV) were included. According to the detection strategy and stringency, the breakpoints in the VIS Atlas database could be classified into three categories, NGS soft-clip reads ≥ 2, NGS soft-clip reads ≥ 3, or non-NGS source (Table 1). Unlike other similar databases, the VIS Atlas database contained 75.04% (47,411/63,179) of human–viral junctional sequences, which we defined as high-resolution VISs. Among them, 46,588 and 823 integration sequences originated from NGS and literature data, respectively. To provide high-quality data, each VIS contained the following information:

**Table 1:** **Summary of items in VIS Atlas database**

**Data source**	**Virus type**	**No. of NGS soft-clip reads**	**No. of accurate integration events**	**No. of integration sequences**	**No. of genotypes**	**No. of diseases**	**No. of publications**	**No. of integration genes**	**No. of experimental methods**
NGS data	HPV	≥ 2	35,562	33,900	38	6	5	13,391	4
		≥ 3	8387	7886	29	4	3	4177	3
	HBV	≥ 2	11,848	11,512	3	2	4	7948	4
		≥ 3	4409	4353	3	2	4	3566	4
	EBV	≥ 2	1418	1176	2	7	6	1459	2
		≥ 3	510	187	2	5	1	129	1
Non-NGS data	HPV	–	583	583	8	5	22	554	17
	HBV	–	13,768	240	7	2	29	7331	13
Total	–	–	63,179	47,411	47	16	38	15,521	23

*Note*: VIS, virus integration site; NGS, next-generation sequencing; HPV, human papillomavirus; HBV, hepatitis B virus; EBV, Epstein–Barr virus.

#### Basic information

**Figure 2:**
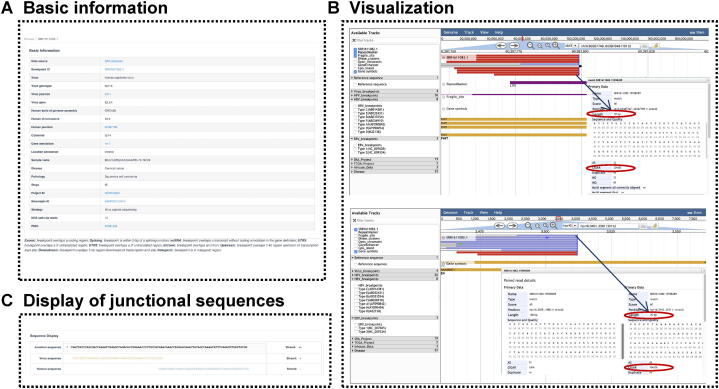
**Three-level integration data** **A.** Basic information. **B.** Manual visualization of supporting reads for NGS-derived computational breakpoints in VIS Browser. This function was built based on the JBrowse genome browser, which was equipped with the human genome (GRCh38), related multi-omics tracks, and all needed virus genomes. SRR1611082.1 (Breakpoint ID), one HPV16 integration site in *FHIT* is given as an example. The top is the mapping view of soft-clip reads in *FHIT*, below is that in the HPV16 genome within the 101-bp window around the integration sites. The supported read SRR1611082.15598289 (Read ID) is 100 bp in length with 32 bp mapped to the human genome and 70 bp to the virus genome (2-bp MH shared by human and virus). **C.** Display of junctional sequences. Chr, chromosome.

This content consisted of integration information and sample metadata. The integration information included virus type, genotype, accurate integration positions in both the human and virus genomes, integration genes and their cytobands in the human genome (provided by ANNOVAR, version 2017-07-17), and integration genes in the virus genome (annotated according to the PaVE database by Perl script). The metadata contained clinical disease type, pathology, stage, integration detection strategy, and publication information (Figure 2). In addition, for 48,828 NGS computational breakpoints, we offered the NGS soft-clip reads, which is a common evaluation index of quality and confidence for VISs [17]. In some reports, only 1 [16], [33], [34] or 2 [32], [35] soft-clip reads are enough for sensitive detection, whereas 3 are high-quality cut-off in most reports [21], [36]. Here, we set the filter options for users to display their interested VISs by choosing NGS soft-clip reads in two different stringencies, ≥ 2 (sensitive mode) or ≥ 3 (confident mode), according to their own needs [32], [34], [35], [37].

#### NGS reads

Each VIS from the NGS data source is accompanied by a link that could visualize the raw mapping results of supported NGS soft-clip reads (Figure 2B). The visualization not only displays the supported reads at related breakpoint positions in both human and virus genomes, but also provides reads ID, mapping length, quality, and other information by clicking on target reads, helping users examine the confidence of each breakpoint (Figure 2).

#### Integration patterns

Our database provided numerous high-resolution junctional sequences, which play an important role in the sentence in analyzing integration patterns. As an advantage, at the bottom of the detailed page for each human–virus junction sequence, we further displayed MH-mediated patterns [21], [22], [23] (Figure S1A) and synthesis-dependent MH-mediated end joining (SD-MMEJ) patterns [38] in 10-bp flanking length (Figure S1B).

### Database usage

**Figure 3:**
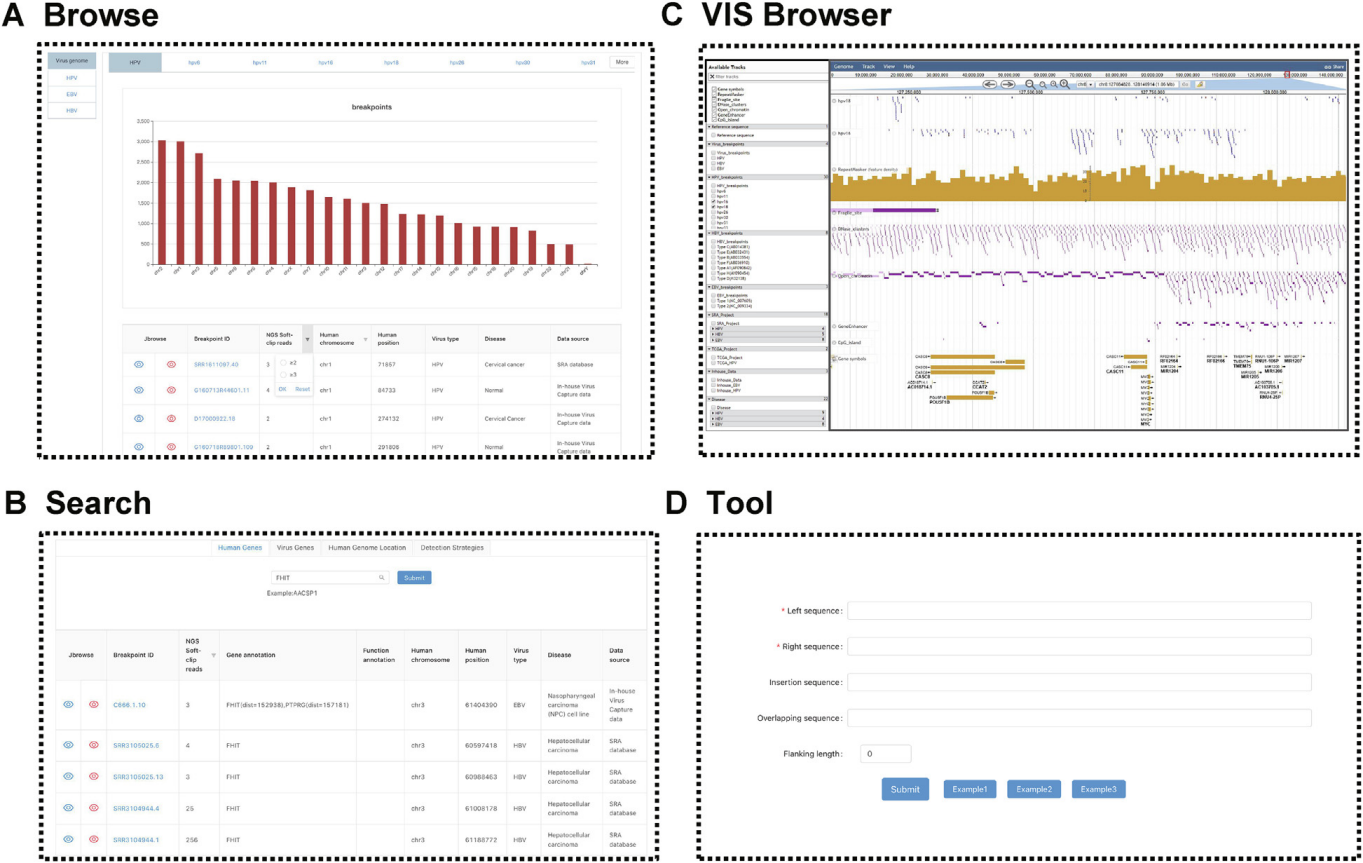
**Main features of theVIS Atlas database** **A.** Browse. On this page, all VISs could be browsed by four main categories, including human genome, virus genome, data source, and disease. **B.** Search. This module allowed users to search VISs by human genes (gene symbol), virus genes, human genomic location (GRCh38), and detection strategies. **C.** VIS Browser. This function was built based on JBrowse genome browser and was equipped with all needed human genome (GRCh38) and virus genome. On this page, users could view breakpoint profiles classified by virus genotypes, data sources, and diseases. **D.** Tool. This tool is aimed to illustrate MH-mediated patterns for fusion sequences.

The web-based interface of VIS Atlas can be freely accessed at http://www.vis-atlas.tech/, and allows users to browse, search, visualize, analyze, and download our integration data **(**Figure 3).

In the Browse module, all VISs in the VIS Atlas database could be browsed by four main categories, including the human genome, virus genome, data source, and disease (Figure 3A). Meanwhile, the Search function is provided for users to choose VISs of interest in four ways, including human genes (supported by gene symbol), virus genes, human genomic location (GRCh38.p12), and detection strategies (Figure 3B). In either Browse or Search module, breakpoint lists could be furthermore filtered by human chromosomes, virus types, and NGS reads.

To display the genome content for a single VIS, we developed a comprehensive genome browser, VIS Browser*,* which was equipped with human genome sequences (UCSC GRCh38.p12 built), human gene annotations (UCSC source), RepeatMasker (UCSC source), fragile sites (UCSC source), DNase clusters (UCSC source), open chromatin (ENCODE source), gene enhancer (UCSC source), CpG island (UCSC source) tracks [39], [40], [41], [42], [43], virus genome sequences (38 HPV, 7 HBV, and 2 EBV genotypes), and virus gene annotations. Furthermore, we clustered breakpoints into blocks and colored them by unique integration times (blue: < 3 times; red: ≥ 3 times) to exhibit the integration hotspots in both human and virus genomes. Besides, we also categorized the visualization according to different virus types, virus genotypes, data sources, and diseases to help study the association between genome functions and different virus integration features (Figure 3C). To our knowledge, this is the first specialized genome browser to help comprehensively explore virus integration local genomic information in both human and virus genomes.

**Figure 4:**
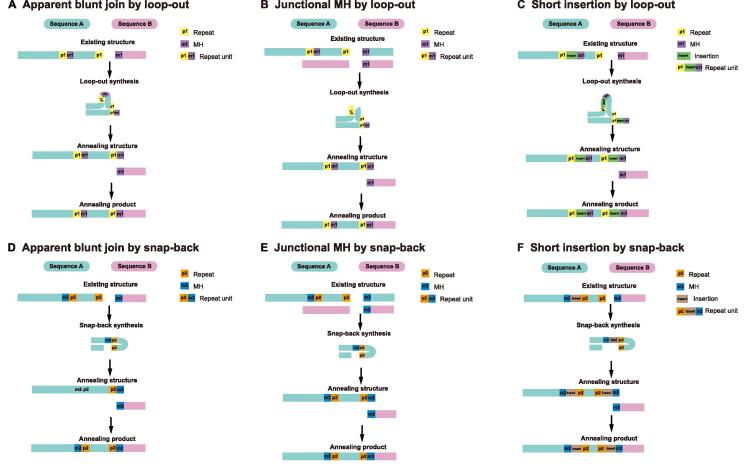
**Illustration of SD-MMEJ repair pathways** **A.** Apparent blunt join products of loop-out modes. **B.** Junctional MH products of loop-out modes. **C.** Short insertion products of loop-out modes. **D.** Apparent blunt join products of snap-back modes. **E.** Junctional MH products of snap-back modes. **F.** Short insertion products of snap-back modes. SD-MMEJ, synthesis-dependent microhomology-mediated end joining.

In addition, high-resolution human–virus junctional sequences could help explore the integration patterns of double-strand DNA viruses. Based on the results analyzed by VIPA, we discovered that a certain quantity of breakpoints may be generated from MH-mediated patterns [44]. For this reason, we also embedded a Tool module (implemented in Perl program language) to calculate and display the MH-mediated patterns (Figure 3D). For instance, the SD-MMEJ pathway could create the MH overhang by synthesis, explaining the highly error-prone essence of the MMEJ pathway [38], [45]. In SD-MMEJ patterns, MHs are synthesized after primers annealing to the upstream identical/complementary bases by loop-out (Figure 4A–C) or snap-back (Figure 4D–F) mode, and then the final end-join process is completed by the annealing of synthesized MH overhangs (Figure 4). Therefore, SD-MMEJ could produce not only junctional MH (overlapping sequence near the junction) (Figure 4C and F) but also the apparent blunt join (direct join with no overlapping sequence near the junction) (Figure 4A and D) and short insertion (unknown sequence near the junction) (Figure 4B and C) products. Based on the aforementioned characteristics, our Tool module could calculate the SD-MMEJ patterns via searching the primers and MHs. Besides, we encourage users to apply other models of virus integration mechanisms in our data to test their own integration theories.

### Data summary

**Figure 5:**
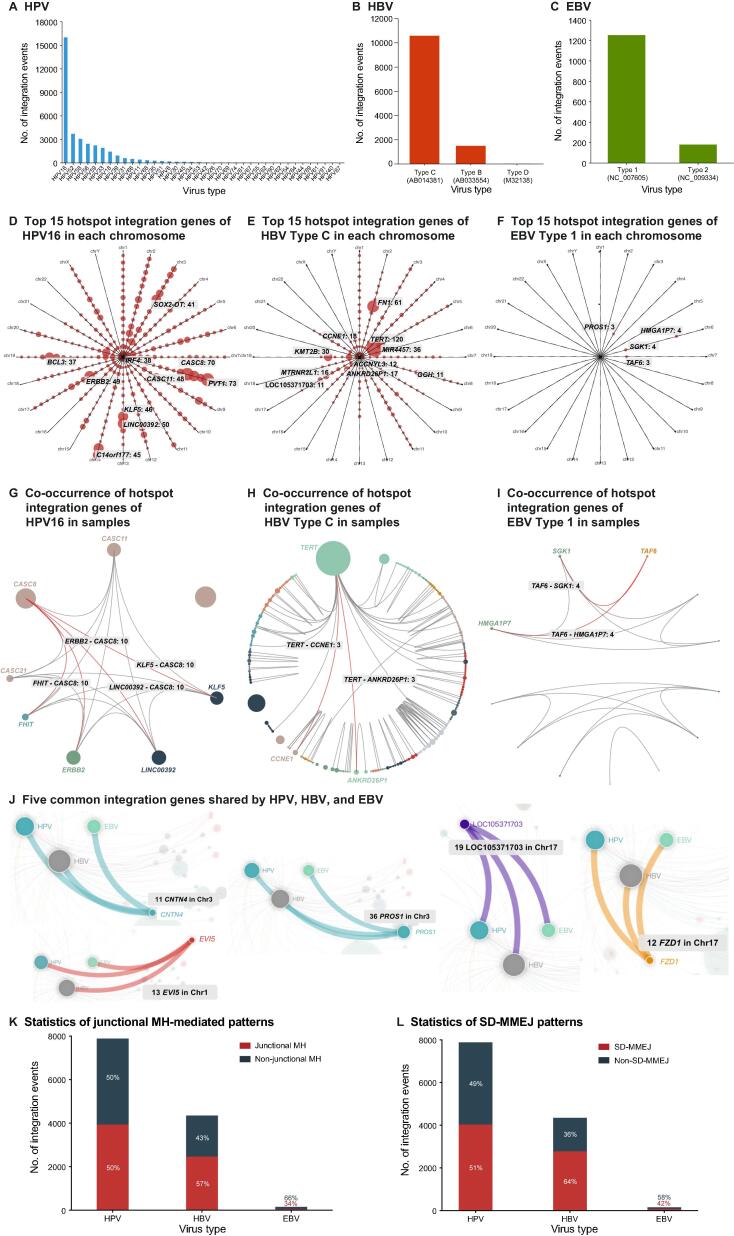
**Statistics of VIS Atlas database** **A.** The breakpoint distribution of HPV genotypes. **B.** The breakpoint distribution of HBV genotypes. **C.** The breakpoint distribution of EBV genotypes. **D.** The top 15 hotspot integration genes of HPV16 in each chromosome. Clock arms and points represent chromosomes and genes, respectively. **E.** The top 15 integration hotspot genes of HBV Type C in each chromosome. **F.** The top 15 hotspot integration genes of EBV Type 1 in each chromosome. **G.** The co-occurrence of hotspot integration genes of HPV16 in samples. The co-existing relationship of integration genes is illustrated by connected lines between gene points. **H.** The co-occurrence of hotspot integration genes of HBV Type C in samples. **I.** The co-occurrence of hotspot integration genes of EBV Type 1 in samples. **J.** The common hotspot integration genes shared by HPV, HBV, and EBV. **K.** The integration events in junctional MH-mediated patterns were summarized in three viruses. **L.** The integration events possessing SD-MMEJ patterns were summarized in three viruses.

The breakpoint distributions of virus genotypes reflect the differences in integration risk. For example, HPV16, HBV Type C (AB014381), and EBV Type 1 (NC007605) were the most high-risk genotypes and had 16,024 (45.06%), 10,451 (88.21%), and 1245 (87.80%) integration events (NGS soft-clip reads ≥ 3), respectively (Figure 5A–C). For integration hotspots of three main virus genotypes, we removed duplicate VISs and summarized the integration gene frequencies (restricted to 500-kb distance). We displayed the top 15 hotspot genes in each chromosome and provided selection options for the unique integration time (at least 3, 5, 10, 15, and 20). When NGS soft-clip reads ≥ 3 and unique integration times ≥ 3, the top ten hotspot genes for HPV16 were *PVT1* (n = 73), *CASC8* (n = 70), *CASC11* (n = 48), *LINC00392* (n = 50), *ERRB2* (n = 49), *KLF5* (n = 46), *C14orf177* (n = 45), *SOX2-OT* (n = 41), *IRF4* (n = 38), and *BCL3* (n = 37) (Figure 5D). HBV Type C had a strong integration preference for chromosome 5 [*TERT* (n = 120) and *MIR4457* (n = 36)], chromosome 19 [*KMT2B* (n = 30) and *CCNE1* (n = 18)], and chromosome 2 [*FN1* (n = 61)] (Figure 5E). EBV Type 1 had four hotspot integration genes, *SGK1* (n = 4), *HMGA1P7* (n = 4), *PROS1* (n = 3), and *TAF6* (n = 3) (Figure 5F).

Furthermore, the hotspot integration genes co-existed in samples, implying their potential biomedical importance. We summarized the aforementioned correlation of hotspot integration genes for HPV16, HBV Type C, and EBV Type 1 genotypes, and provided two explorative options: (1) at least 3, 5, 10, 15, and 20 integrated samples for hotspot genes, and (2) at least 3, 5, 10, 15, and 20 samples for co-existing relationship. We found that for HPV16, each pair among the seven hotspot integration genes (*CASC8*, *CASC11*, *CASC21*, *LINC00392*, *KLF5*, *FHIT*, and *ERBB2*) co-existed in more than 10 samples (NGS soft-clip reads ≥ 3 and integration hotspots in ≥ 15 samples) (Figure 5G; Table S2). For HBV Type C, inter-chromosomally, *TERT* had co-existing integration relationship with *CCNE1* and *ANKRD26P1* in 3 samples (NGS soft-clip reads ≥ 3 and integration hotspots in ≥ 3 samples) (Figure 5H; Table S3). Similarly, for EBV Type 1, the hotspot integration gene *TAF6* co-existed with *SGK1* and *HMGA1P7* in 4 samples (Figure 5I; Table S4).

Similarly, the common integration genes among three most prevalent double-stranded DNA viruses may reveal marked biological pathogenesis. We displayed integration genes with ≥ 2 virus types, and provided the selection options of total unique integration times (at least 3, 5, 10, 15, and 20). When NGS soft-clip reads ≥ 3 and total unique integration times ≥ 10, 5 common integration genes (*CNTN4*, *EVI5*, *FZD1*, LOC105371703, and *PROS1*) were found in HPV, HBV, and EBV (Figure 5J; Table S5).

Except for the common integration genes, the VIS Atlas database also focused on the integration patterns shared by three double-strand viruses. Here, we provided a total of 47,411 specific integration sequences for users to test their own algorithm. For instance, we explored and summarized potential MH-mediated integration patterns among three virus types and detailed genotypes, including junctional MH-mediated and SD-MMEJ patterns. When NGS soft-clip reads ≥ 3, we found that 50% HPV, 57% HBV, and 66% EBV integration sequences had ≥ 2 bp junctional MHs (Figure 5K). Meanwhile, SD-MMEJ patterns could be discovered in 51% HPV, 64% HBV, and 58% EBV integration sequences (Figure 5L). These results indicated that the VIS Atlas database could provide a platform for the research of oncogenic virus integration patterns and mechanisms.

## Discussion

In this study, we constructed a database of NGS breakpoints from the three most prevalent oncogenic viruses, HPV, HBV, and EBV. We developed an integration calculation algorithm to increase the detection sensitivity and at the same time guarantee the confidence of soft-clip reads in two ways. (1) Initially chimeric mapping results against the mixed reference of specific virus genotype and human were re-checked by mapping to specific virus genotype and human alone via BWA-MEM to avoid align errors. (2) When all soft-clip reads were clustered by genome coordinate, BLASTN was utilized to check the human and virus positions of reads to exclude inaccurate results. We believe the strategies mentioned above could achieve reliability and sensitivity in detecting HPV, HBV, and EBV integration, and could construct a consistent breakpoint database based on different NGS data sources.

As most current genome browsers have no double-strand virus genome and annotation configuration, we developed VIS Browser as the first customized genome browser to visualize and explore NGS computational VISs. The VIS Browser could help users not only to visualize both collected and their own computational results, but also to understand the potential impact of local genomic context on viral integration. For instance, open chromatin and DNase clusters reflect the more accessible genome regions [43], [46]. Fragile sites and DNA repeats may explain the generation of double-strand break and transfer mechanisms of viral DNA [41], [42], [47]. CpG islands and enhancers can give hints about the downstream biological function of the VISs [39], [40].

Furthermore, we focused on the genotype-based VISs in Browse, Search, Visualization, and Statistic modules. The virus genotypes showed differences in integration frequencies, human hotspots, and associated disease stages. Our results support the viewpoint that the prevention and treatment strategies for oncogenic viruses should be based on virus genotypes and even sub-genotypes [48].

The understanding of oncogenic viral integration mechanisms could provide important information for both preventive and therapeutic strategies against the corresponding virus persistent infections and their related cancers [49]. Unlike retroviruses which produce the integrase to facilitate the viral insertion, most DNA viruses have not been discovered to possess viral protein similar to integrase. Therefore, the mechanism mediating the integration process still remains elusive. Here with amounts of integration sequences from both NGS and literature data, we constructed the most comprehensive database for single-base resolution sequences of viral–human fusional DNA scars, exploring the potential DNA repair mechanisms for viral insertional mutagenesis. We provided a total of 47,411 integration sequences for users to test their own algorithms. For instance, we summarized potential MH-mediated integration patterns among three virus types. When NGS soft-clip reads ≥ 3, 50% HPV, 57% HBV, and 66% EBV integration sequences had ≥ 2-bp junctional MHs (Figure 5K). These results indicate that the VIS Atlas database could provide a platform for the research of oncogenic virus integration patterns.

As NGS becomes the most popular detection approach for VISs, the need to develop a computational VIS database is required. The VIS Atlas database based on integration sequences from NGS data sources makes the study of integration patterns possible. Additionally, we will continue to maintain and improve our database in the future by following strategies: (1) adding breakpoints of other viruses, such as molluscum contagiosum virus (MCV) and herpes simplex virus (HSV); (2) expanding more disease types and samples; and (3) acquiring accurate VISs and sequences by taking virus integration heterogeneity into account.

As the first universal resource for NGS integration breakpoints, the VIS Atlas database is expected to help promote research into oncogenic virus integration mechanisms during carcinogenesis and the development of preventive and therapeutic strategies for virus-related cancers.

## Data availability

The VISs identified in this study with their annotations and sequences can be directly downloaded at http://www.vis-atlas.tech/.

## Competing interests

The authors have declared no competing interests.

## CRediT authorship contribution statement

**Ye Chen:** Investigation, Validation, Methodology, Writing – review & editing. **Yuyan Wang:** Software, Formal analysis, Methodology, Writing – review & editing. **Ping Zhou:** Methodology, Investigation, Funding acquisition. **Hao Huang:** Methodology, Investigation, Formal analysis. **Rui Li:** Investigation, Validation, Resources. **Zhen Zeng:** Investigation, Validation, Resources. **Zifeng Cui:** Writing – original draft. **Rui Tian:** Resources, Methodology. **Zhuang Jin:** Formal analysis. **Jiashuo Liu:** Data curation. **Zhaoyue Huang:** Data curation. **Lifang Li:** Visualization. **Zheying Huang:** Visualization. **Xun Tian:** Conceptualization, Project administration, Funding acquisition. **Meiying Yu:** Resources, Methodology, Project administration. **Zheng Hu:** Conceptualization, Methodology, Project administration, Funding acquisition, Supervision, Writing – original draft, Writing – review & editing. All authors have read and approved the final manuscript.

## Supplementary material

Supplementary Figure S1 **The integration patterns for two breakpoints in VIS Atlas database A.** The microhomology-mediated patterns. Microhomologies were indicated in dashed Box. **B.** The SD-MMEJ patterns.

Supplementary Table S1 **Comparison of VIS Atlas and VISDB**

Supplementary Table S2 **Co-occurrence relationship of high-frequency integration genes for HPV16 with soft-clip read ≥ 3 and integrated samples ≥ 10**

Supplementary Table S3 **Co-occurrence relationship of high-frequency integration genes for Type C genotype with the criteria of soft-clip read ≥ 3 and integrated samples ≥ 3**

Supplementary Table S4 **Co-occurrence relationship of high-frequency integration genes for Type 1 genotype with the criteria of soft-clip read ≥ 3**

Supplementary Table S5 **Common high-frequency integration genes shared by three viruses with the criteria of NGS soft-clip reads ≥ 3**
