# Supplementary material for: VIS Atlas: A Database of Virus Integration Sites in Human Genome from NGS Data to Explore Integration Patterns
Source: Genomics Proteomics Bioinformatics. 2023 Feb 16;21(2):300–10. doi: 10.1016/j.gpb.2023.02.005 (PMC10626058; doi:10.1016/j.gpb.2023.02.005)
Supplement: Supplementary Figure S1 — The integration patterns for two breakpoints in VIS Atlas database A. The microhomology-mediated patterns. Microhomologies were indicated in dashed Box. B. The SD-MMEJ patterns. [file mmc1.pdf]

A

Sequence Display

**Junction sequence**	> AAAAAAGGAAAGTGTGCCTATTCTTTTATGCATTGTATTTTCAACATATCAAACTTATTGGGGTCAGGTAAATGTATTCTAAATACCCTGTATTGTAATC	**Strand:** +/-
**Virus sequence**	CAAACTTATTGGGGTCAGGTAAATGTATTCTAAATACCCTGTATTGTAATC	**Strand:** -
**Human sequence**	AAAAAAGGAAAGTGTGCCTATTCTTTTATGCATTGTATTTTCAACATATCA	**Strand:** +
**MH patterns**	TTCAACATAT.CA.AACTTATTGG	junctional microhomology/Snap-back
	TTCAACATAT.CA.AACTTATTGG	junctional microhomology/Loop-out
	TTCAACATAT.CA.AACTTATTGG	junctional microhomology/Loop-out

Microhomologies

B

Sequence Display

**Junction sequence**	> CTCTCTCTTAGACAGATAGACACACACACACACACACGCACACATCCAGTTCCAGTTTCTGCAGGTGTTGGAGGCTGCAATACAGATGGGTCAGTGAAAG	**Strand:** +/-
**Virus sequence**	TCCAGTTTCTGCAGGTGTTGGAGGCTGCAATACAGATGGGTCAGTGAAAG	**Strand:** -
**Human sequence**	CTCTCTCTTAGACAGATAGACACACACACACACACACGCACACATCCAGT	**Strand:** +
**MH patterns**	CACATCCAGT-TCCAGTTTCT	apparent blunt join/Loop-out

The end-join between human and virus sequence was displayed in three ways (junctional microhomology within dots; dash for apparent blut join; lowercase for short insertion). The repeat, including primers (red) and microhomologies (blue), was underlined.
